# Tracheotomy in Diphtheria

**Published:** 1890-02-08

**Authors:** 


### SURGERY.

### TRACHEOTOMY IN DIPHTHERIA.

Professor Henoch gives in the *Deutsch. Med. Wochensch.*, 1889, No. 44, some statistics as to the course and terminations of the cases of diphtheria that came under his care in the Charité Hospital at Berlin during the years 1886-7, so far as the disease occurred in children. Of these, there were 192; in 110 it was throughout limited to the pharynx, extending in the remaining 82 to the larynx, or, as he calls it, "passing into croup." In 12 of these no operation was attempted, the patients being already pulseless, or presenting evidence of septic poisoning, and these all died. Of the 70 on whom tracheotomy was performed, only nine (13 per cent.) recovered. This very unfavourable result, contrasting strongly with the successes achieved by others, is partly to be attributed to the fact that his patients were drawn from the very poorest of the population, and the lowest quarters of the city, but far more to the distinction which he draws between "diphtheritic and idiopathic croup," for of 36 cases of the latter in which he operated, 24 (66 per cent) recovered—a very large proportion indeed. Of the 110 cases of diphtheria uncomplicated with croup, only 32 died.

But why talk of croup at all? a term that means, or may mean, anything, and leads to endless confusion. Diphtheritic processes may commence in the pharynx, the larynx or the nose, and they may (theoretically) extend from the one to either or both of the other mucous tracts. In practice, however, laryngeal diphtheria does not spread, for the simple reason that, unless the attack be mild and short, it kills the child by suffocation. Diphtheria primarily laryngeal is the more favourable for operation, though very fatal otherwise; but when the laryngeal disease is secondary, or the consequence of extension from the pharynx, the surgeon finds his patient already exhausted and poisoned by the primary or pharyngeal disease. The nasal form is very fatal, because insidious and mostly implicating the larynx. Indeed, it is often not recognised as such until laryngeal symptoms appear. These are probably included among Henoch's cases of "idiopathic croup."

He generally observed albumenuria during the illness, beginning usually between the third and fourth day; but nephritis, as a sequela of the disease, which it mostly is in scarlatina, he saw only twice. In only one was there articular rheumatoid disease, and this recovered in a few days without febrile disturbance.

